# Intraoperative Indocyanine Green Fluorescence Angiography for Real-Time Validation of Carotid Artery Patency Following Resection of Advanced Carotid Body Tumors

**DOI:** 10.7759/cureus.103783

**Published:** 2026-02-17

**Authors:** Fernando Dip, Rene Aleman, Raul Peceros, Sergio Ferreyra Fernandez, Mariano Norese

**Affiliations:** 1 Department of General Surgery, University of Buenos Aires, Buenos Aires, ARG; 2 Heart, Vascular &amp; Thoracic Institute, Cleveland Clinic Florida, Weston, USA

**Keywords:** carotid body tumor, carotid resection, fluorescence angiography, indocyanine green, indocyanine green fluorescence angiography, perfusion assessment, vascular reconstruction

## Abstract

Carotid body tumors (CBTs) are rare paragangliomas arising at the carotid bifurcation, with Shamblin III lesions posing substantial operative risk due to circumferential arterial encasement. When arterial resection and reconstruction are required, reliable confirmation of carotid perfusion is essential. This is the case of a 24-year-old male patient with a right-sided Shamblin III CBT managed with preoperative embolization, segmental internal fluorescence-guided carotid artery resection, and end-to-end reconstruction. Intraoperative indocyanine green fluorescence angiography (ICG-FA) demonstrated immediate, homogeneous perfusion across the reconstructed segment, with no delay or focal hypofluorescence. The postoperative course was uneventful, with no neurologic or vascular complications. This case illustrates the potential role of intraoperative ICG-FA as a real-time adjunct to confirm carotid patency and perfusion following complex vascular tumor resection.

## Introduction

Carotid body tumors (CBTs) are uncommon neuroendocrine paragangliomas originating from chemoreceptor tissue at the carotid bifurcation, accounting for 0.6% of head and neck neoplasms [1,2]. Surgical complexity is commonly stratified using the Shamblin classification, with type III tumors characterized by circumferential carotid encasement and a higher likelihood of arterial injury, resection, or reconstruction [3,4]. In cases requiring carotid sacrifice or reconstruction, preservation of cerebral perfusion is paramount. Traditional intraoperative assessment tools, including visual inspection, palpation, or Doppler interrogation, provide indirect feedback and may not reliably detect subtle anastomotic compromise. Although digital subtraction angiography offers definitive evaluation, its intraoperative use is limited by invasiveness and logistical constraints. 

Indocyanine green fluorescence angiography (ICG-FA) enables near-infrared visualization of intravascular flow and tissue perfusion in real time. Its utility is well established in cerebrovascular bypass surgery, reconstructive microsurgery, and oncologic perfusion assessment [5,6]. Nevertheless, its application in CBT surgery involving carotid reconstruction remains sparsely reported. The authors herein describe a rare case of Shamblin III CBT in a young adult in which intraoperative ICG-FA was implemented to confirm carotid patency following segmental resection and end-to-end reconstruction. 

## Case presentation

**Figure 1:**
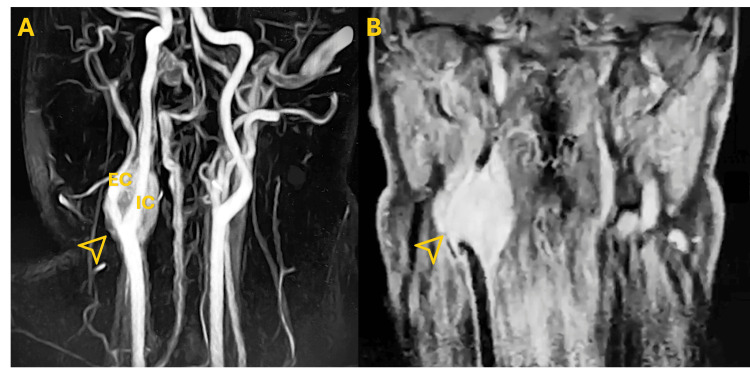
Right-Sided Carotid Body Tumor (CBT) at the Carotid Bifurcation Imaging findings demonstrated a right-sided CBT at the bifurcation of the internal and external carotid arteries. (A) Contrast-enhanced magnetic resonance angiography showed a hypervascular mass at the carotid bifurcation, causing characteristic splaying of the internal carotid artery (IC) and external carotid artery (EC), consistent with a CBT. (B) Corresponding soft-tissue imaging confirmed a well-defined enhancing lesion at the same level. Yellow arrowheads indicate the tumor margins in both panels.

A 24-year-old male patient presented with a progressively enlarging, painless right cervical mass. Duplex ultrasonography revealed a hypervascular lesion at the carotid bifurcation. Contrast-enhanced computed tomography and digital subtraction angiography confirmed a CBT with circumferential encasement of the carotid axis, consistent with a Shamblin III lesion (Figure 1). A carotid occlusion test demonstrated preserved neurologic function and hemodynamic stability, supporting tolerance to temporary carotid clamping upon performance of surgical resection.

**Figure 2:**
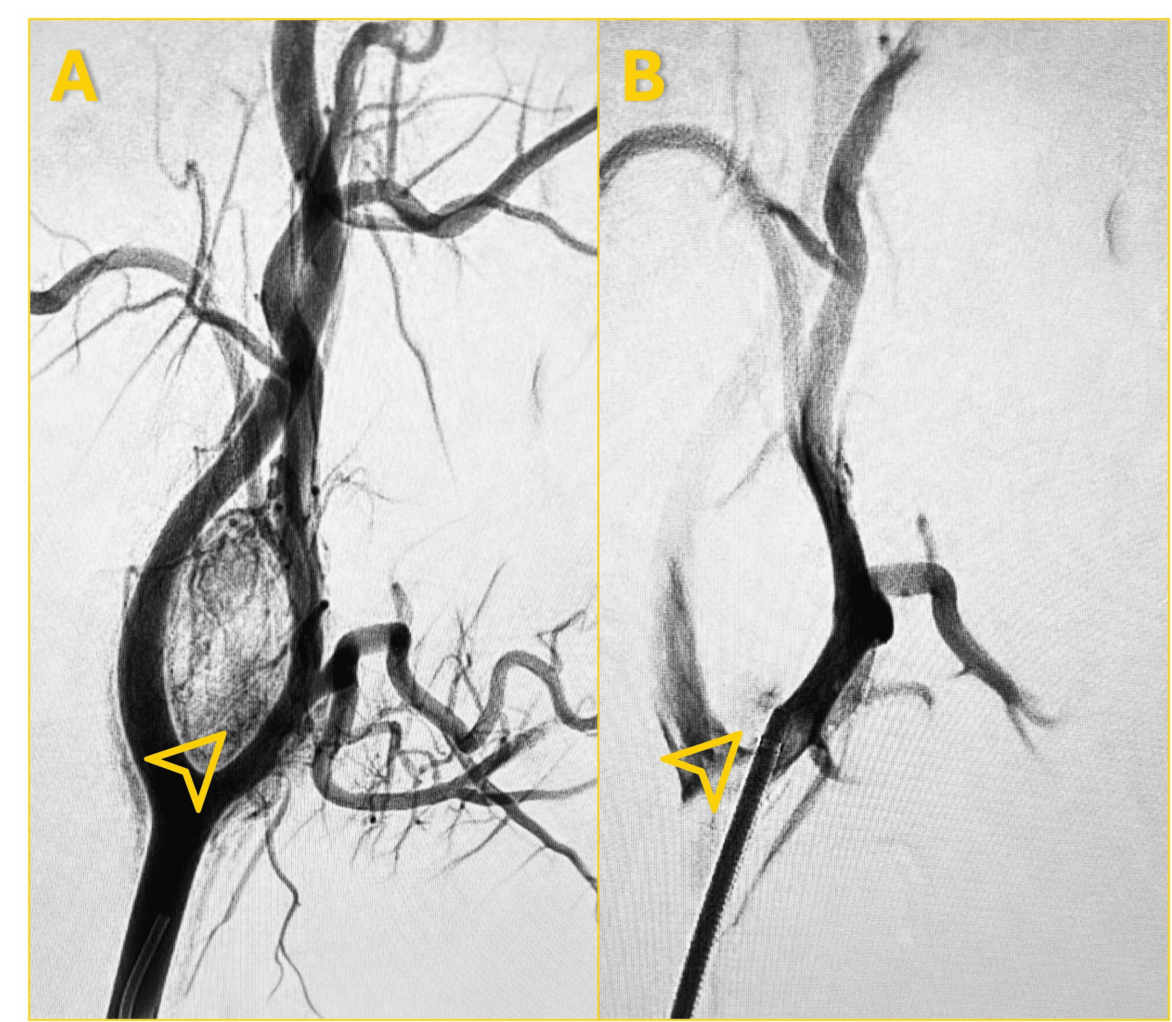
Selective Embolization of a Carotid Body Tumor (CBT) Angiography demonstrated preoperative selective embolization of tumor-feeding vessels supplying the right-sided CBT. (A) Pre-embolization angiography showed a densely hypervascular tumor blush at the carotid bifurcation with prominent arterial feeders (arrowhead). (B) Post-embolization angiography obtained four days prior to surgery demonstrated a marked reduction in tumor vascularity with effective devascularization and absence of residual tumor blush (arrowhead).

Selective coil embolization of tumor-feeding vessels was performed four days prior to surgery to reduce vascularity. Post-embolization angiography confirmed effective devascularization (Figure 2). The patient was provided with verbal and written consent prior to intervention. 

Surgical technique 

Upon induction of general anesthesia, a transverse cervical incision was performed with subplatysmal flap elevation and anterior mobilization of the sternocleidomastoid muscle. A firm, approximately 4-cm hypervascular mass was identified enveloping the internal and external carotid arteries and carotid bulb. Proximal and distal vascular control of the common, internal, and external carotid arteries was obtained. 

Subadventitial dissection allowed mobilization of approximately 80% of the tumor. Dense adherence between the tumor and the internal carotid artery precluded complete separation without compromising oncologic margins. To achieve complete resection, a short segment of the internal carotid artery, approximately 2 mm in length, was excised en bloc with the tumor. 

**Figure 3:**
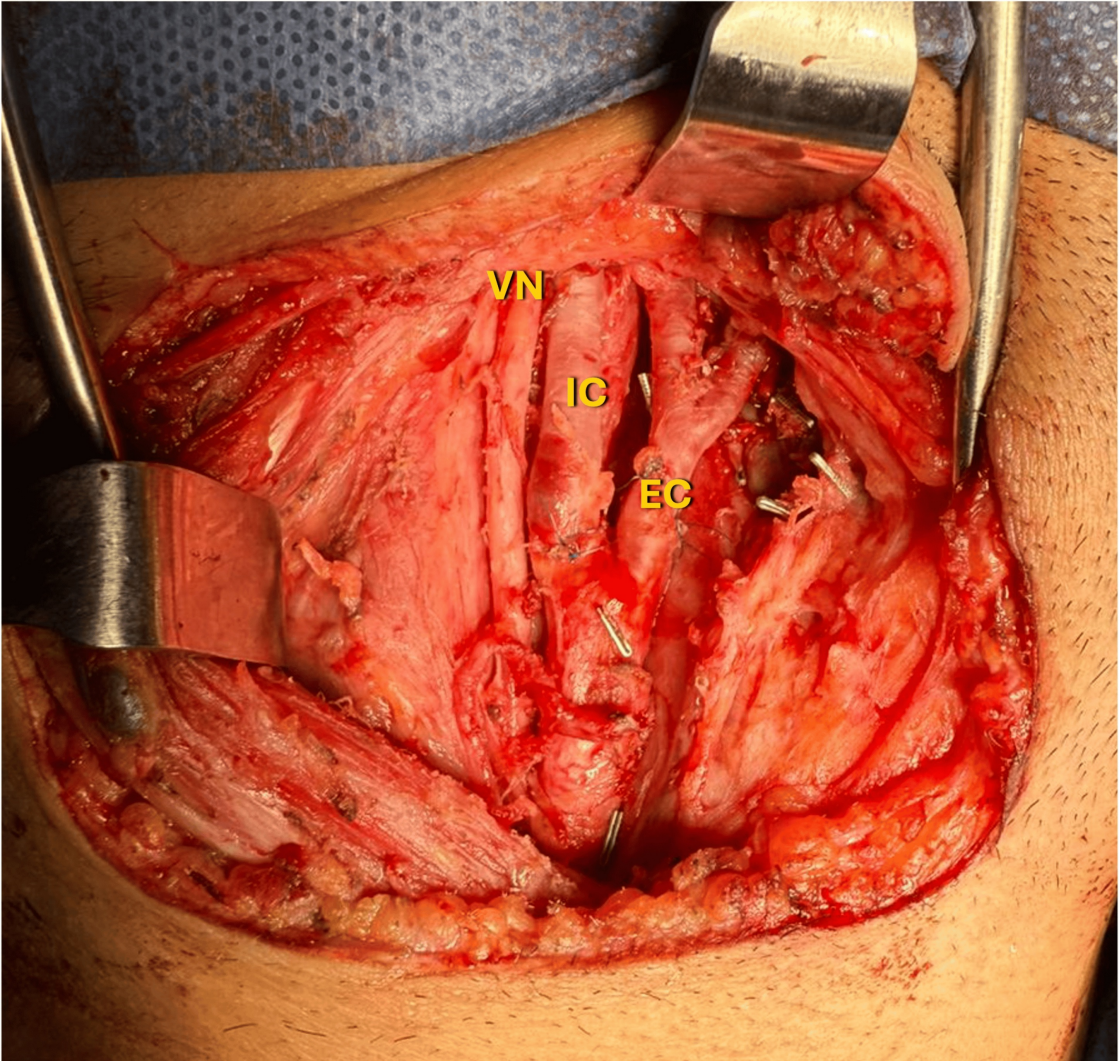
Intraoperative Exposure During Carotid Body Tumor (CBT) Resection Intraoperative view of a right-sided CBT; Subadventitial dissection allowed partial mobilization. VN: vagus nerve; IC: internal carotid artery; EC: external carotid artery

After systemic heparinization, the artery was clamped for 15 minutes and reconstructed with a beveled end-to-end, termino-terminal anastomosis using standard microsurgical technique (Figure 3). Upon completion, intraoperative perfusion was assessed using intravenous ICG-FA. Immediate and homogeneous fluorescence was observed across the reconstructed segment, with preserved anterograde flow and no focal delay or hypofluorescence, confirming patency and anastomotic integrity (Video 1). 

**Video 1:** Intraoperative ICG-FA After Carotid Reconstruction Intraoperative ICG-FA demonstrated assessment of carotid artery integrity following tumor resection and arterial reconstruction. After peripheral administration of 1 mL (2.5 mg) of reconstituted ICG, near-infrared imaging showed immediate and homogeneous fluorescence across the reconstructed segment, confirming preserved anterograde flow and anastomotic patency. ICG-FA: indocyanine green fluorescence angiography

**Figure 4:**
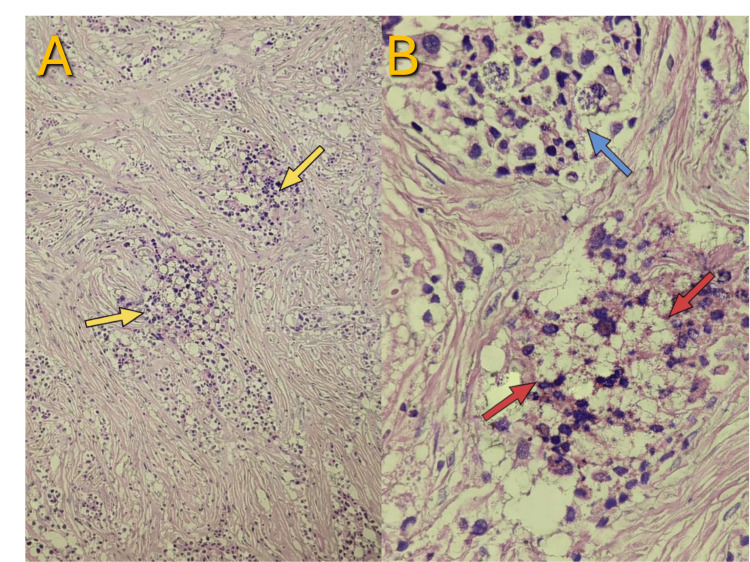
Histopathologic Features of Carotid Body Tumor (CBT; Paraganglioma) Light microscopy of the resected CBT demonstrated characteristic histopathologic features of paraganglioma. (A) Low-power (4× objective) hematoxylin and eosin (H&E) section showed a poorly circumscribed paraganglioma composed of nests and diffuse sheets of chromaffin cells embedded within a fibrosclerotic stroma (yellow arrows), consistent with a CBT (Shamblin III). Areas of stromal fibrosis and hemorrhage were evident, without vascular or lymphatic invasion. (B) High-power (20× objective) H&E view demonstrated tumor cells with granular (“salt-and-pepper”; blue arrow) chromatin, amphophilic cytoplasm, and mild nuclear anisocytosis arranged predominantly in a diffuse pattern with focal alveolar (Zellballen) architecture. Foci of infarct-type necrosis (approximately 10%; red arrows), attributed to prior embolization, were present. No atypical mitotic figures were identified. Overall findings were consistent with an extra-adrenal paraganglioma of the carotid body, completely resected.

The patient’s postoperative course was uneventful, and was discharged on postoperative day 1. No neurologic deficits, cranial nerve dysfunction, or ischemic complications were observed in the immediate postoperative period and at the one-month follow-up. Histopathology confirmed a carotid body paraganglioma with negative margins (Figure 4). 

## Discussion

Shamblin III CBTs represent the most surgically demanding subset of paragangliomas due to circumferential carotid encasement, loss of the subadventitial dissection plane, and frequent need for arterial sacrifice or reconstruction. Multiple surgical series have demonstrated that these tumors are associated with higher rates of vascular injury, cranial nerve morbidity, and perioperative neurologic complications compared with Shamblin I and II lesions. Presentation in young adults is uncommon and further underscores the importance of preserving long-term carotid patency and neurologic function [3,4,7].

Preoperative embolization remains an important adjunct in the management of highly vascular CBTs. By reducing arterial inflow, embolization has been associated with improved operative visualization, decreased blood loss, and safer dissection, particularly in large or high-grade tumors [4,8]. In the present case, angiographic confirmation of effective devascularization likely facilitated vascular exposure and enabled controlled proximal and distal carotid control prior to tumor mobilization. 

Despite advances in embolization and surgical technique, complete subadventitial dissection is not always achievable in Shamblin III tumors. Dense adherence or partial invasion of the arterial wall may require limited carotid resection to achieve negative margins. Short-segment resection with primary end-to-end reconstruction is a well-established vascular oncologic strategy that preserves native arterial geometry and avoids prosthetic material when feasible, particularly in young patients. When performed with meticulous microsurgical technique, termino-terminal reconstruction has demonstrated favorable long-term patency and low rates of restenosis [9].

A critical intraoperative challenge in these cases is the reliable confirmation of carotid perfusion following reconstruction. Traditional assessment methods, including visual inspection, palpation, Doppler interrogation, stump pressure measurements, or transcranial Doppler, provide indirect or surrogate information and may fail to detect subtle anastomotic compromise, focal narrowing, intimal flap formation, or early thrombosis. While intraoperative digital subtraction angiography offers definitive assessment, it introduces additional invasiveness, radiation exposure, contrast administration, and workflow disruption. 

ICG-FA offers a complementary solution by providing real-time, non-invasive visualization of intravascular flow. ICG rapidly binds plasma proteins and emits near-infrared fluorescence, allowing direct assessment of vessel patency and flow dynamics at the operative field. Its utility is well established in cerebrovascular bypass surgery, reconstructive microsurgery, and oncologic perfusion assessment [5,6]. However, its application in carotid reconstruction following CBT resection has been infrequently reported, despite the clear theoretical advantages in this setting. 

In the present case, ICG-FA demonstrated immediate, homogeneous fluorescence across the reconstructed internal carotid artery, with preserved anterograde flow and no segmental hypofluorescence or delay. This provided objective confirmation of anastomotic integrity and vessel vitality prior to wound closure. Such real-time feedback is particularly valuable in Shamblin III tumors, where arterial manipulation is extensive, and the margin for error is narrow. Crucially, ICG-FA added negligible operative time, rescinded the need for the required arterial puncture, and avoided radiation exposure. 

Several limitations warrant consideration. As a single-case report, the findings cannot be generalized, and no causal inference regarding outcome improvement can be made. Indocyanine green fluorescence angiography is qualitative and does not provide absolute flow quantification, pressure gradients, or predictive information regarding long-term patency. Interpretation remains operator-dependent, and standardized thresholds for abnormal fluorescence have not been established. Accordingly, ICG should be viewed as an adjunct rather than a replacement for established vascular assessment techniques.

Nonetheless, the value of ICG lies in its ability to rapidly exclude critical intraoperative complications, such as gross flow limitation or anastomotic failure, at a decision-making juncture when revision remains feasible. Emerging literature in vascular and endovascular surgery supports the broader integration of real-time optical perfusion assessment to enhance procedural safety and intraoperative confidence [10,11]. Future multicenter studies may help define standardized interpretive criteria, reproducibility, and integration with other monitoring modalities in complex carotid surgery. 

This case highlights the convergence of meticulous preoperative planning, selective arterial resection to ensure oncologic completeness, microsurgical reconstruction, and real-time perfusion validation. Intraoperative ICG-FA represents a practical and informative adjunct in complex CBT surgery requiring vascular reconstruction, with the potential to enhance intraoperative decision-making and surgical safety without adding meaningful risk. 

## Conclusions

Shamblin III CBTs are rare and pose significant surgical challenges. When arterial resection and end-to-end reconstruction are required, confirmation of carotid perfusion is critical. Contextually, intraoperative ICG-FA provided rapid, objective, and non-invasive confirmation of carotid patency following reconstruction. While limited to adjunctive use, ICG-FA can potentially enhance intraoperative confidence and safety in complex vascular tumor resections involving carotid reconstruction.

## References

[1] Shamblin WR, ReMine WH, Sheps SG, Harrison EG Jr (1971). Carotid body tumor (chemodectoma). Clinicopathologic analysis of ninety cases. Am J Surg.

[2] Wang SJ, Wang MB, Barauskas TM, Calcaterra TC (2000). Surgical management of carotid body tumors. Otolaryngol Head Neck Surg.

[3] Siedek V, Waggershauser T, Berghaus A, Matthias C (2009). Intraoperative monitoring of intraarterial paraganglioma embolization by indocyaningreen fluorescence angiography. Eur Arch Otorhinolaryngol.

[4] Sajid MS, Hamilton G, Baker DM (2007). A multicenter review of carotid body tumour management. Eur J Vasc Endovasc Surg.

[5] van der Bogt KE, Vrancken Peeters MP, van Baalen JM, Hamming JF (2008). Resection of carotid body tumors: results of an evolving surgical technique. Ann Surg.

[6] van der Mey AG, Jansen JC, van Baalen JM (2001). Management of carotid body tumors. Otolaryngol Clin North Am.

[7] Arya S, Rao V, Juvekar S, Dcruz AK (2008). Carotid body tumors: objective criteria to predict the Shamblin group on MR imaging. AJNR Am J Neuroradiol.

[8] Robertson V, Poli F, Hobson B, Saratzis A, Ross Naylor A (2019). A systematic review and meta-analysis of the presentation and surgical management of patients with carotid body tumours. Eur J Vasc Endovasc Surg.

[9] Tange FP, van den Hoven P, van Schaik J (2024). Near-infrared fluorescence imaging with indocyanine green to predict clinical outcome after revascularization in lower extremity arterial disease. Angiology.

[10] Vaassen HG, Lips DJ, Geelkerken RH, Wermelink B (2025). Quantitative intra-arterial fluorescence angiography for direct monitoring of peripheral revascularization effects. J Vasc Surg Cases Innov Tech.

[11] Pal R, Lwin TM, Krishnamoorthy M (2023). Fluorescence lifetime of injected indocyanine green as a universal marker of solid tumours in patients. Nat Biomed Eng.

